# Postoperative intermittent pneumatic compression for preventing venous thromboembolism in Chinese lung cancer patients: a randomized clinical trial

**DOI:** 10.1186/s12959-023-00498-z

**Published:** 2023-05-10

**Authors:** Jingyao Li, Aihong Huang, Zhaojie Han, Yi Zhou, Meng Tang, Wei Wu, Shixin Zhang, Kelong Liao, Yihui Xie, Qiao Chen, Xinliang Zou, Shuai Liu, Shuaixiang Gao, Junlong Ren, Qingyuan Xu, Xi Liu, Yi Liao, Tao Jing, WenFeng Tan, Yang Qiu, Haidong Wang

**Affiliations:** 1 Department of Thoracic Surgery, Southwest Hospital, Army Medical University, (Third Military Medical University), Chongqing, 400038 China; 2 Department of Vasculocardiology, Southwest Hospital, Army Medical University, (Third Military Medical University), Chongqing, China; 3 Department of Neck and Chest Surgery, Affiliated Hospital of Sergeant School of Army Medical University, Shijiazhuang, China

**Keywords:** Lung cancer, Venous thromboembolism (VTE), Intermittent pneumatic compression (IPC), Catheter-related thrombosis (CRT), Perioperative management

## Abstract

**Background:**

Postoperative lung cancer patients belong to the high-risk group for venous thromboembolism (VTE). The standardized preventive measures for perioperative VTE in lung cancer are not perfect, especially for the prevention and treatment of catheter-related thrombosis (CRT) caused by carried central venous catheters (CVCs) in lung cancer surgery.

**Patients and methods:**

This study included 460 patients with lung cancer undergoing video-assisted thoracic surgery (VATS) in our center from July 2020 to June 2021. Patients were randomized into two groups, and intraoperatively-placed CVCs would be carried to discharge. During hospitalization, the control group was treated with low-molecular-weight heparin (LMWH), and the experimental group with LMWH + intermittent pneumatic compression (IPC). Vascular ultrasound was performed at three time points which included before surgery, before discharge, and one month after discharge. The incidence of VTE between the two groups was studied by the Log-binomial regression model.

**Results:**

CRT occurred in 71.7% of the experimental group and 79.7% of the control group. The multivariate regression showed that the risk of developing CRT in the experimental group was lower than in the control group (Adjusted RR = 0.889 [95%CI0.799–0.989], *p* = 0.031), with no heterogeneity in subgroups (P for Interaction > 0.05). Moreover, the fibrinogen of patients in the experimental group was lower than control group at follow-up (*P* = 0.019).

**Conclusion:**

IPC reduced the incidence of CRT during hospitalization in lung cancer patients after surgery.

**Trial registration:**

No. ChiCTR2000034511.

**Supplementary Information:**

The online version contains supplementary material available at 10.1186/s12959-023-00498-z.

## Introduction

The occurrence of lung cancer represents 11.6% of all cancer cases, and the mortality rate is 18.4% of cancer deaths, ranking first among all malignancies [1]. Lung cancer-related venous thromboembolism (VTE), a complication, is the second leading cause of death other than malignancy itself [2, 3]. Among cancer patients, those who underwent significant surgeries and were kept bedridden long-term were at a greater risk of VTE [4, 5]. Deep vein thrombosis (DVT) and pulmonary embolism (PE) are two clinical manifestations of VTE at different sites and stages [6]. They can occur in various parts of the body, especially the deep veins in the lower limbs, and DVT is the primary source of thromboembolism in PE [7].

Aggressive perioperative prophylaxis is vital to reduce the incidence of VTE in lung cancer patients [8, 9]. The most commonly used thoracic surgery type is applying a modified Caprini score scale for risk stratification [10]. For patients with a moderate risk score (5–8) of Caprini and at low risk of bleeding, LMWH for 7–10 d along with mechanical prophylaxis was recommended; for patients with a high-risk score (≥ 9), and a low risk of bleeding, the use of LMWH for 30 d along with mechanical prophylaxis was recommended [10–12]. The patients after lung cancer surgery were generally in medium and high-risk groups, so perioperative prevention was necessary [13]. Additionally, in thoracic surgery, central venous catheters (CVCs) were placed into the patient's internal jugular and subclavian for volume resuscitation, intravenous drug delivery, and hemodynamic monitoring [14]. The most common complications of CVCs placement were catheter occlusion and catheter-related thrombosis (CRT) [15]. After venous congestion, the structure of endothelial cells gradually deteriorates and releases plasminogen activator to produce VTE [16]. IPC has been shown to prevent deep vein thrombosis, mainly due to the periodic emptying of deep veins caused by pneumatic sleeves compression [17]. Compression also has a systemic effect on blood coagulation and fibrinolytic potential, stimulating fibrinolysis [18].

According to the thrombus-related guidelines [9, 12, 19, 20], we conducted a prospective randomized clinical trial to analyze VTE incidence after postoperative LMWH + IPC antithrombotic therapy in the Chinese population and its safety.

## Methods

### Study design and patient population

The study was a randomized controlled trial, in which all eligible subjects were randomly assigned to the control group (LWMH) and the experimental group (LWMH + IPC) after signing the informed consent forms. The ethics committee has approved this study of the First Affiliated Hospital of the Army Medical University. (Ethics approval number: KY2020110, China Clinical Trial Registration Center Registration No. ChiCTR2000034511).

Inclusion criteria: 18–65; without a history of thrombosis; diagnosed with lung cancer by pathology and imaging; without a history of other cancers; without a history of chemotherapy; about to undergo conventional thoracotomy or thoracoscopic surgery. Exclusion criteria: acute venous thrombosis; deep thrombophlebitis; erysipelas; acute cardiopulmonary disease, pulmonary edema, arrhythmia, and unstable hypertension; pacemaker users; high risk of bleeding; contraindicated with unfractionated heparin or low molecular weight heparin.

### Randomization and trial treatment

Random sequences were generated by random number tables and sealed in consecutive envelopes by a dedicated researcher. Envelopes were opened in chronological order of subject enrollment. Data collectors and examining physicians were blinded to the random assignment.

CVCs with a diameter of 2.4 mm, and a three-lumen, three-way valve catheter (SPECATH, Foshan, China) were placed by an anesthesiologist. Postoperative maintenance was performed by a professional thoracic surgery care team following standard procedures until discharge. IPC (PTQ-2, Chongqing Pengtai Medical Device Co., Ltd.) was applied two hours after the operation, with an airbag placed in the patient's thigh and calf. The tightness was adjusted to accommodate only one finger, and the pressure was set at 60 ~ 90 mmHg (1 mmHg = 0.133 kPa). The pressure was gradually adjusted from low to an effective range that lasted for 30 min, and this was performed twice daily until one week after the surgery.

The investigators evaluated the inclusion and exclusion criteria to meet the requirements of this trial. After signing their informed consent forms, the subjects were randomized into control and experimental groups (1:1) by a randomized digital table.

Control group (LMWH): 4000 units of LMWH (Enoxaparin Sodium Injection, Sanofi) were given 12 h before surgery, and then 4000 units once a day until the patient was discharged.

Experimental group (LMWH + IPC):4000 units of LMWH (Enoxaparin Sodium Injection, Sanofi) were given 12 h before surgery, and 4000 units once a day until the patient was discharged. The IPC was also applied to the patients for the prevention of VTE.

A patient was observed in the intensive care unit for at least one day after surgery, and then transferred to the general ward after his/her situation was allowed. Both groups were encouraged to move gently in bed or out of bed according to their postoperative recovery.

### Temporary anticoagulation adjustment

High-dose thromboprophylaxis (enoxaparin 40 mg bid) was performed in morbidly obese patients with a weight > 100 kg and BMI ≥ 40 kg/m^2^ and without renal insufficiency or other contraindications. Enoxaparin at 30 mg qd was administered to patients with low weight (female < 45 kg; male < 57 kg) or a high risk of severe renal dysfunction (CrCl < 30 mL/min).

### Surveillance and follow-up

The follow-up time of this study is before discharge and one month after hospital discharge. If the patients were diagnosed with VTE before discharge, consultation with the multidisciplinary team were arranged first. Patients were given follow-up treatment according to their condition. In general, the patient would take rivaroxaban tablets (10 mg) 1/day for one month after discharge. D-dimer, prothrombin time (PT), activated partial thromboplastin time (APTT), fibrinogen (FIB), thrombin time (TT), platelet (PLT), ultrasound, and leg circumference measurements were conducted on the follow-up.

### Outcome measures

The leading observational indicators of this study were the incidence of postoperative VTE in the two groups and the occurrence of side effects. To evaluate the effectiveness of IPC in the prevention of VTE after lung cancer surgery.

All patients were examined for coagulation-related tests (D-dimer, PT, APTT, FIB, TT, PLT) and vascular ultrasound before surgery and seven days after surgery. By vessel ultrasound, thrombosis was monitored as venous diameter thickening; partial/complete blocking in the lumen by a solid echo; lumen disappearing by compression; abnormal Doppler signals such as accelerating, weakening, or disappearance of blood flow in the proximal part (downstream) when squeezing the distal limbs; loss of continuity of blood flow in distal portion and weakened or lost response to Valsalva movement; and side branch cycle formation.

The leg circumference was measured the day before surgery and every day after surgery until discharge. Measurement was as follows: observation of consistency in skin color and temperature of both lower limbs, and whether there was swelling and pain. Perimeters of both lower limbs (thigh circumference: 10 cm above the patella; calf circumference: 10 cm under the tibial nodules) were measured daily with tape to understand the swelling of the affected limbs. For patients with possible VTE, the motor and sensation of both lower limbs should be examined, and strip-shaped foreign bodies with tenderness should be searched for.

Another objective of this study was to examine the risk factors for perioperative VTE in lung cancer patients. A Caprini risk assessment model was used to evaluate the risk in subjects, and a clinical quantitative score grading system for risk assessment of VTE was preliminarily established. Patients were scaled with the modified Caprini assessment on the day before and after surgery (Supplementary Table 1).

### Statistical considerations

The occurrence of VTE in this experiment was composed of DVT and CRT. The incidence of DVT was approximately 1.6% [21] according to previous literature reports, and the incidence of CRT was about 66.0% [22]. The incidence of DVT was much lower than that of CRT, so the sample size was estimated here with CRT as the main outcome variable. The incidence of thrombosis applying IPC + LWHM was 48% [23] lower compared with LWMH alone. Considering the application of the Z-Test with Unpooled Variance, 95% power, bilateral 5% significance level of (α), a sample size of 191 for each group, and a 20% lost-to-follow-up rate, a total of 460 subjects were required.

Data analysis was performed using R software (Version 4.1.3). Median and interquartile spacing were used for describing continuous variables, and frequency was used for categorical variables. Continuous type variables were all nonnormal by the Shapiro-Wilks test, and differences between groups were indicated by Wilcoxon's rank sum tests. Categorical variables were tested by the chi-square test. A multiple collinearity test was performed between the variables. Variable screening for the final regression model was performed using the change-in-estimate (CIE). This means after culling an independent variable from a multiple regression model, considered deleting the variable if the effect on the β value of the target factor doesn’t exceed 10%. Due to the large incidence of outcomes in this randomized controlled study, the Log-binomial regression model was chosen to avoid false estimation of RR. We performed subgroup analyses by grouping according to the median value of the selected continuous variable.

## Results

### Patient distribution

**Fig. 1:**
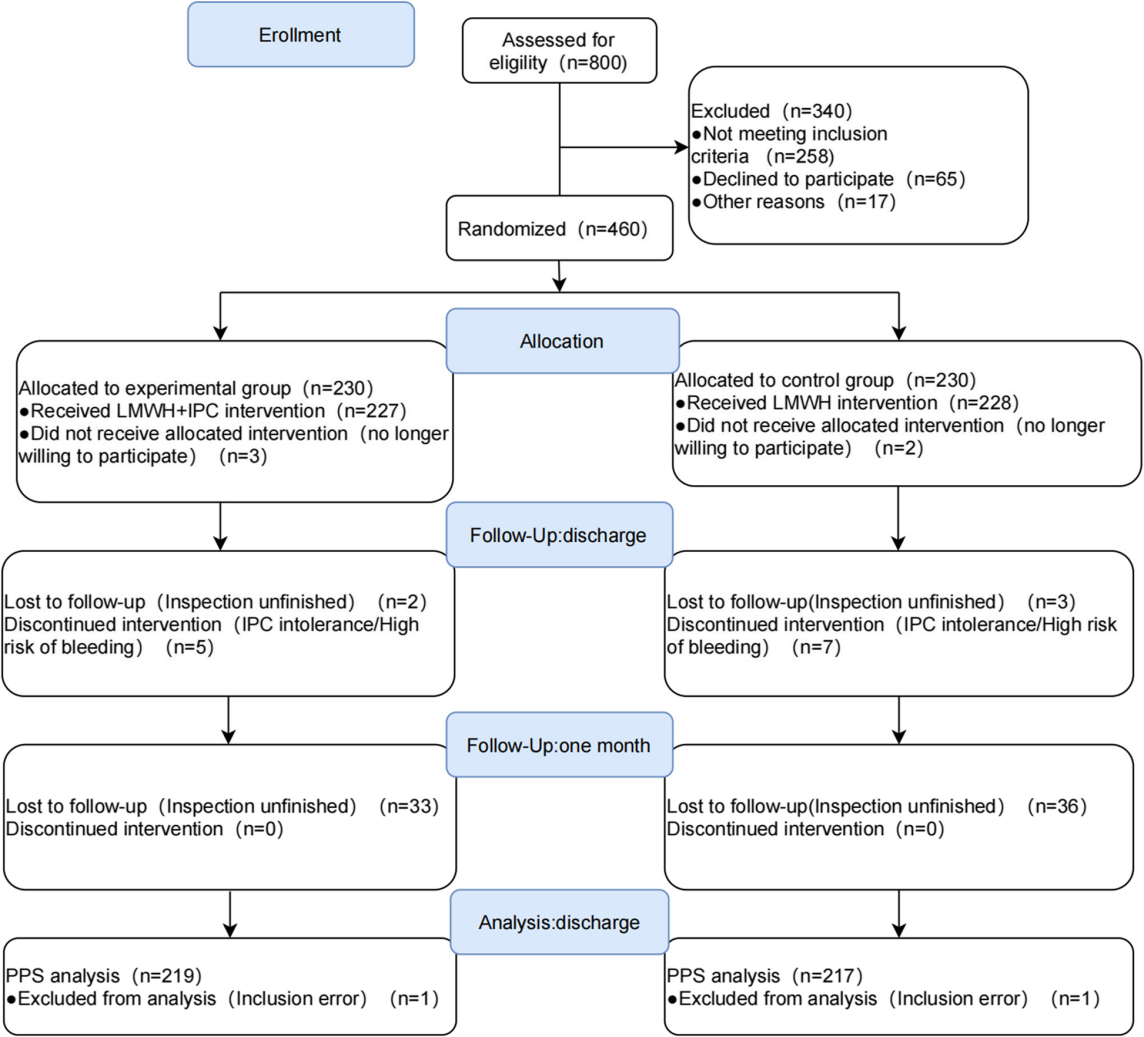
Participant flow chart

A total of 460 patients were randomly assigned to the experimental group (*n* = 230) and the control group (*n* = 230). The first follow-up was performed at hospital discharge: 11 patients withdrew from the experimental group due to automatic withdrawal, IPC intolerance, high risk of postoperative bleeding, and incomplete examination; 13 patients withdrew from the control group. In this study, the data of the experimental group (*n* = 219) and the control group (*n* = 217) at discharge were analyzed. At the second follow-up, which was one month after discharge, 33 patients in the experimental group and 36 in the control group refused to be reviewed. The flow chart was shown in Fig. 1.

The baseline characteristics of the subjects in both groups: gender, active smoking status, cancer pathological type, lung cancer staging, age, BMI, preoperative D-dimer, PT, APTT, FIB, TT, PLT, perimeter, catheter to vein ratio (CTVR), surgical method and preoperative Caprini were not significantly different (*p* > 0.05) (Table 1).

**Table 1:** The baseline information

	**Control group** **(*N* = 217)**	**Experimental group** **(*N* = 219)**	***P* value**
Gender: male	86 (39.6%)	75 (34.2%)	0.244
Smoking status: yes	57 (26.3%)	53 (24.2%)	0.619
Pathological type: adenocarcinoma	200 (92.2%)	198 (90.4%)	0.516
Lung cancer stage: early	192(88.5%)	201(91.8%)	0.320
Age (years)	53 [48, 57]	52 [47, 57]	0.078
BMI (kg/m2)	23.5 [21.6, 25.7]	23.4 [21.4, 25.0]	0.285
D-dimer^a^(mg/L)	0.18 [0.12, 0.27]	0.17 [0.12, 0.26]	0.740
PT ^a^ (s)	10.3 [10.0, 10.7]	10.3 [9.9, 10.7]	0.331
APTT ^a^(s)	26.8 [25.5, 28.1]	26.6 [25.4, 27.9]	0.250
FIB^a^ (g/L)	2.62 [2.27, 3.01]	2.59 [2.33, 3.00]	0.792
TT^a^(s)	17.9 [17.4, 18.5]	18.0 [17.4, 18.5]	0.737
PLT^a^(10^9/L)	200 [167, 237]	202 [170, 246]	0.456
Perimeter ^a^ (cm)	35.65 [34.10, 37.40]	35.58 [33.90, 37.21]	0.754
CTVR(%)	24.0 [20.0, 27.6]	24.0 [21.1, 27.7]	0.279
Surgical type^b^: Lobectomy	169 (77.9%)	180 (82.2%)	0.229
Wedge Resection	39 (18.0%)	27 (12.3%)	
Segmentectomy	9 (4.15%)	12 (5.48%)	
Lymph node dissection^b^	176(81.1%)	189(86.3%)	0.142
Pleural adhesions^b^	50 (23.0%)	50 (22.8%)	1
Caprine ^c^(points)	7 [7, 8]	7 [7, 7]	0.257

Values were presented as n (%) or median [IQR]. ^a^ indicated preoperative results; ^b^ indicated the intraoperative results; ^c^ indicated postoperative results

*Abbreviations*: *BMI* Body mass index, *PT* Prothrombin time, *APTT* Activated partial thromboplastin time, *FIB* Fibrinogen, *TT* Thrombin time, *PLT* Platelet, *CTVR* Tube diameter/internal venous internal diameter, *CVC* Central venous catheter

### Primary analysis

All patients underwent thoracoscopic lung cancer surgery, and six of them were converted to an intraoperative thoracotomy. Specifically, 80% of the patients underwent lobectomy,20% underwent wedge or pulmonary segment resection, 83.7%underwentregional lymph node dissection, and 22.9% required intraoperative pleural adhesion management. The median of CVCs placement in both groups was 6.00 days [5.00; 7.00] (*P* = 0.63). Specifically, the median in lobectomy was 6.00 days [5.00; 7.00], wedge resection was 4.00 days [3.00; 6.00], and segmentectomy was 4.00 days [4.00; 5.00]. Performing one-way log-binomial regression on the catheterization duration of all patients, RR 95%CI = 1.02 [1.01, 1.04], *P* value < 0.001. DVT was found by vascular ultrasound, specifically, three (1.4%) in the control group and one (0.5%) in the experimental group. CRT was produced in 173 (79.7%) control patients and 157 (71.7%) patients in the experimental group (*p* = 0.051). The multivariate Log-binomial regression showed that the risk of developing CRT in the experimental group was lower than in the control group (Adjusted RR = 0.889 [95% CI: 0.799–0.989], *p* = 0.031) (Table 2). One month after discharge, a total of 359 patients returned to the hospital for review, and 24 patients still had CRT, including 14 patients in the control group and 10 patients in the experimental group.

**Table 2:** Intervention RR was compared with the adjust RR

RR	95% CI	P value	Adjust RR	95% CI	*P* value
0.899	0.808–1.001	0.051	0.899	0.799–0.989	0.031

### Exploratory analyses

**Fig. 2:**
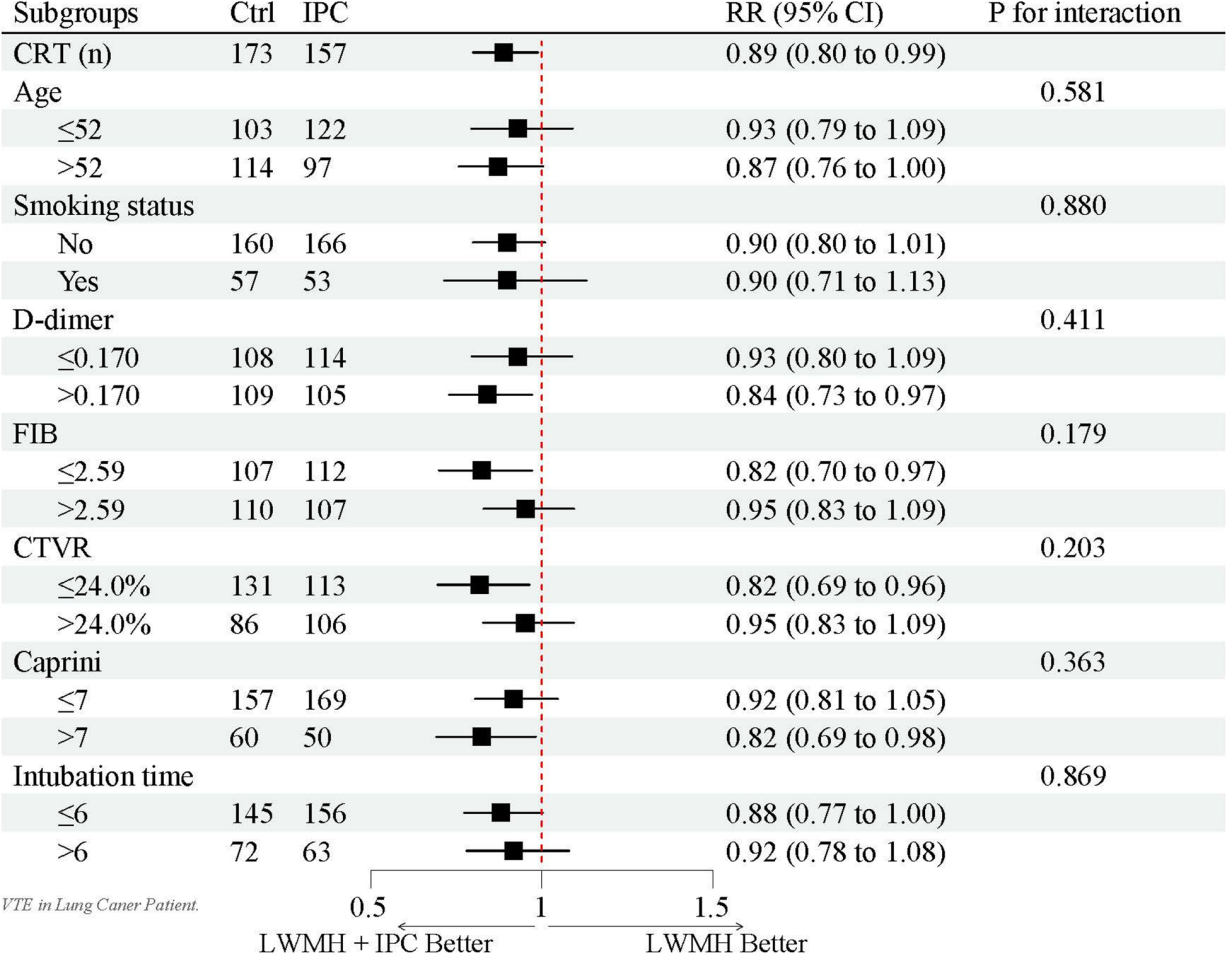
Interactive item subgroup analysis

Multicollinearity tests suggested a possible collinearity between preoperative and postoperative Caprini scores and no collinearity among the remaining variables. Considering the actual situation, the postoperative Caprini variable is retained when the regression equation is constructed. The preoperative TT is adjusted in the log-binomial regression model as a confounding factor according to the CIE method. According to the literature review, the four subgroups were set to D-dimer, FIB [24], CTVR [25], Caprini score [26], and age, smoking status, and extubation time were set to be the three exploratory subgroups for the interactive item subgroup analysis. The subgroup analysis showed that with D-dimer > 0.17 mg/L, FIB < 2.59 g/L, CTVR < 24% and Caprini score > 7, the effect of CRT reduction by using IPC might be more significant, but the interaction term showed no significant difference in treatment effect between the groups (P for Interaction > 0.05). The findings were robust in the subgroup analyses (Fig. 2).

**Fig. 3:**
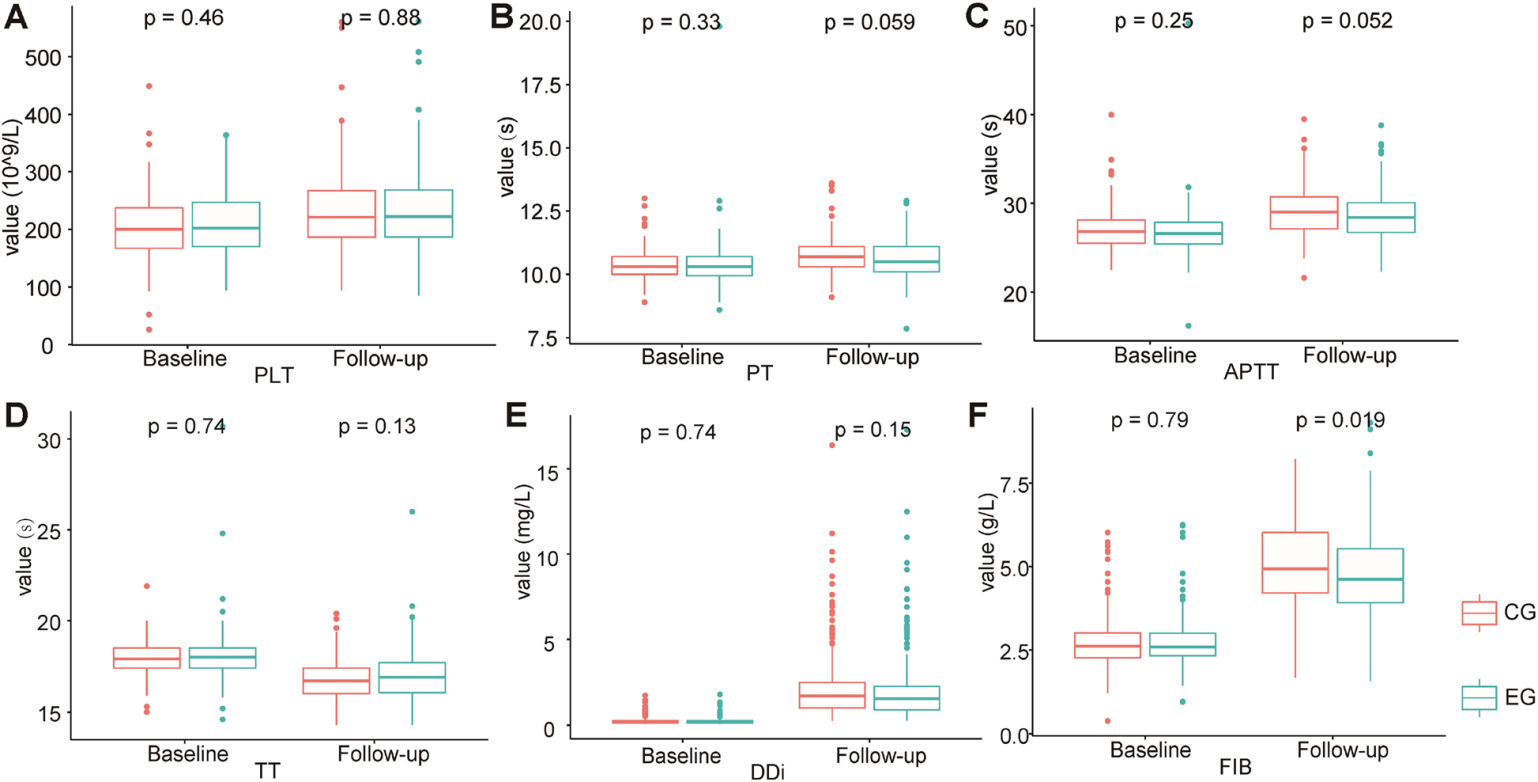
Differences in PLT, PT, APTT, TT, D-dimer, and FIB between the control group and the experimental group at baseline and follow-up (discharge). Abbreviations: EG, experimental group; CG, control group

Furthermore, there were no significant differences in D-dimer, PT, APTT, TT, PLT, or leg circumference between the two groups at the follow-up, but in FIB was statistically significant (*P* = 0.019) (Fig. 3).

## Discussion

This is the first prospective, randomized, controlled clinical trial in the Chinese population using LMWH + IPC antithrombotic therapy during the perioperative phase of lung cancer, especially for CRT caused by CVCs placement in lung cancer surgery, which is an easily neglected post-operative risk factor.

Although Chinese medical staff have a positive attitude toward the clinical application of mechanical prevention, the relevant knowledge and standardized operation need to be strengthened [27]. There are no relevant studies on whether IPC reduces CRT, and there are no specific guidelines or consensus for automated prevention, and the existing relevant policies and agreements are not detailed enough for mechanical prevention, leading to deficient feasibility. The guidelines recommended using IPC for more than 18 h per day and an extension for completely inactive patients [28]. In consideration of patients’ tolerance, machine availability, and the number of nurses, IPC was given twice a day for 30 min and maintained until the patient was discharged. We found that patients in the experimental group may had a lower CRT incidence rate than those in the control group (*P* = 0.051). The incidence of DVT in the lower limbs in the LMWH group was 1.4%, which was consistent with the results obtained by Joseph et al. [21] This was much lower than the incidence in patients with lung cancer without any prevention (7.3–13.9%) [29, 30]. Therefore, for lung cancer patients with a perioperative Carprini score of medium–high risk, the early use of LMWH could reduce VTE occurrence. However, there was no significant difference in lower limb DVT incidence between the LMWH + IPC and the LMWH groups, and the effect of preventing VTE with LMWH in the absence of CVCs alone was similar to that of LMWH + IPC.PE is mainly derived from lower limb DVT [6, 7], but there is still a possibility of PE formation from CRT shedding. Although central venous channels were considered in the Caprini score, they have not attracted enough attention [31]. LMWH was proven ineffective in preventing CRT [32, 33], and we similarly found that CRT developed up to 75.7% of patients at discharge (71.7% in the LMWH + IPC group and 79.7% in the LMWH group). The incidence of CRT in this study was higher than that in the previous literature (27–66%)) [22], probably because 91.3% of the patients enrolled in this study had lung adenocarcinomas. Lung adenocarcinoma is a high-risk factor for CRT in cancer patients [34]. One month after release and oral administration of anticoagulants, 24 patients still had neck thrombosis. For cancer patients, chemotherapy is a high-risk factor for thrombosis [35]. If a patient still has VTE detected before chemotherapy, the chemotherapy will be inevitably affected. Peripherally Inserted Central Catheter (PICC) cannot completely replace CVCs for chemotherapy because it is a recent technique with uncertain results comparing its effectiveness with CVCs [36]. Therefore for patients with thrombosis, thrombosis will be treated before chemotherapy for safety, which may delay the time of chemotherapy.

FIB varied between the two groups (*p* = 0.019), with elevated FIB, blood being hypercoagulable, slower blood flow, and increased risk of thrombosis. IPC can promote the activation of the fibrinolytic system, increase the release of plasminogen and the activity of plasmin, prevent fibrinolytic shutdown commonly seen in postoperative patients [37], and accelerate the dissolution of blood clots, thereby preventing the formation of DVT. The FIB was higher in the control group than in the experimental group, confirming that IPC might stimulate the body to deal with the hypercoagulation state by affecting the blood flow changes through intermittent pressure, which was consistent with the literature [24]. In the exploratory subgroups, although there was no significant difference between subgroups, the effects of CRT reduction by IPC might be more significant in the subgroup with D-dimer > 0.17 mg/L, FIB < 2.59 g/L, CTVR < 24%, and Caprini score > 7. Interaction term subgroup analysis was performed using the median value of each continuous variable as the cutoff value. Although the D-dimer and FIB were not completely consistent with the clinical outliers, the actual situation might be more consistent for the Chinese lung cancer population. The cutoff value of 7 in the Caprini in this study was also consistent with the individualized VTE risk stratification set in the literature [26]. A catheter with a tube diameter of 2.4 mm was used in this study. Since a larger venous diameter indicates a smaller CTVR, IPC pressure promotes blood flow better and produces CRT less likely. Therefore, for patients after lung cancer surgery, special attention should also be paid to the above possible high-risk factors, and we also need to design targeted studies for the above subgroups to confirm. It should be noted that when the CRT is large, thrombus shedding when the catheter is removed may cause PE, which leads to sudden death. In thoracic surgery, CVCs were placed into the patient's internal jugular and subclavian for volume resuscitation, intravenous drug delivery, and hemodynamic monitoring. However, the current guidelines and consensus do not specify the duration of CVCs placement after thoracic surgery, therefore other centers in China perform CVCs extraction based on their own experiences. We removed the CVCs before a patient was discharged. Notably, since the patients underwent different methods of surgeries, the length of hospital stay also varied. For example, a considerable part of our patients underwent surgeries which lengthened the hospital stay, such as lobectomy (80%), regional lymph node dissection (83.7%), and intraoperative pleural adhesion management (22.9%), resulting in longer duration of postoperative recovery in these patients. In this study, the effect of catheterization time on the main effect analysis in both groups was less than 10%, and although it was not included in the final regression equation, a univariate regression analysis of catheterization time for all patients showed statistically significant results (*P* < 0.001), indicating that the probability of CRT events increases with prolonged catheterization time. Therefore, it is safer to remove the catheter as early as possible after surgery. However, for patients who cannot have early extubation due to unstable situations, applying IPC together is beneficial to reduce CRT. It is also suggested that patients with lung cancer should use a catheter with anticoagulant as much as possible. Meanwhile, ultrasound should be checked before discharge to ensure safety.

This study also has some limitations. Due to restrictions in the medical background, there are no corresponding guidelines for the postoperative catheterization time for lung cancer patients. It is necessary to conduct large-scale real-world retrospective or prospective clinical studies to find the optimal time for catheter removal and establish industry standards. This is also the most effective way to prevent CRT. However, in the context where early catheter removal is not possible, IPC prevention can be an effective choice. Furthermore, considering both effectiveness and cost-effectiveness, devices similar to elastic compression stockings should be included in the study for comparison in preventing CRT, in order to find a more convenient and cheaper prevention method. Due to factors such as patient refusal to follow-up or loss of contact, the rate of loss to follow-up after one month is about 15%. Although there are too many confounding factors within one month to facilitate data analysis, it also provides us with more experience for follow-up work in future studies. Developing a mobile follow-up system and providing more help and attention to patients can help reduce the loss to follow-up rate and achieve better follow-up results.

## Conclusion

IPC can reduce the formation of postoperative CRT in lung cancer patients. It is recommended to start LMWH + IPC prophylaxis early in patients with medium–high Caprini risks. The CVCs were suggested to be pulled out as soon as possible after the patient was in stable condition. In the future, it is necessary to explore possible risk factors for the purpose of reducing CRT with accurate experimental designs to find the most suitable VTE prevention method under the realistic situation following thoracic surgery.

## Supplementary Information

**Additional file 1: Supplementary table 1.** Modified Caprini Score Scale for VTE control of lung cancer patientsAQ. 

## Data Availability

All raw data are available upon request from the corresponding author.

## Abbreviations

VTE: Venous thromboembolism
CRT: Catheter-related thrombosis
CVCs: Central venous catheters
VATS: Video-assisted thoracic surgery
LMWH: Low-molecular-weight heparin
PE: Pulmonary embolism
DVT: Deep vein thrombosis
PT: Prothrombin time
APTT: Activated partial thromboplastin time
FIB: Fibrinogen
TT: Thrombin time
PLT: Platelet
CIE: Change-in-estimate
CTVR: Catheter to vein ratio
PICC: Peripherally inserted central catheter
IPC: Intermittent pneumatic compression
