# Supplementary material for: Postoperative intermittent pneumatic compression for preventing venous thromboembolism in Chinese lung cancer patients: a randomized clinical trial
Source: Thromb J. 2023 May 10;21:56. doi: 10.1186/s12959-023-00498-z (PMC10170726; doi:10.1186/s12959-023-00498-z)
Supplement: Supplementary file 1 — Additional file 1: Supplementary table 1. Modified Caprini Score Scale for VTE control of lung cancer patientsAQ. [file 12959_2023_498_MOESM1_ESM.docx]

| **Supplementary table 1. Modified Caprini Score Scale for VTE control of lung cancer patients** | | | | | |
| --- | --- | --- | --- | --- | --- |
| Age 40-59 (y) | 1 | Age 60-74 (y) | 2 | Age 75 (y) | 3 |
| Abnormal pulmonary function | 1 | Central venous access | 2 | History of VTE | 3 |
| Acute myocardial infarction (<1mo) | 1 | Confined to bed (>72 h) | 2 | Family history of VTE | 3 |
| BMI≥30 (kg/m^2^) | 1 | Major open surgery (45 min) | 2 | Chemotherapy | 3 |
| Congestive heart failure (<1mo) | 1 | Present cancer | 2 | Positive anticardiolipin antibody | 3 |
| History of inflammatory bowel disease | 1 | Prior cancer, except nonmelanoma skin | 2 | Positive Lupus anticoagulant | 3 |
| History of prior major surgery (<1 mo) | 1 |  |  |  |  |
| Complications of pregnancy | 1 |  |  | Acute spinal cord injury (<1 mo) | 5 |
| Oral contraceptive use or HRT | 1 |  |  | Major surgery 6 h | 5 |
| Sepsis (<1 mo) | 1 |  |  |  |  |
| Serious acute lung disease (<1 mo) | 1 |  |  |  |  |
| Swollen legs (current) | 1 |  |  |  |  |
| Varicose veins | 1 |  |  |  |  |
| Amount: |  | | | | |
| Low risk (0-4) Medium risk (5-8) High risk (≥9) | | | | | |
